# The Treatment of Bronchitis

**Published:** 1893-08-26

**Authors:** 


### CITY ROAD CHEST HOSPITAL.

### The Treatment of Bronchitis.

In describing treatment as practised at this hospital, it is necessary, for the sake of clearness, to take the subject under the two heads of—(*a*) Acute, and (*b*) Chronic.

(*a*) *Acute Bronchitis.*—The patient on admission is at once put to bed, and as in those cases (the majority) in which dyspnœa is a marked symptom, a bed rest adjusted to a moderate height is used. Instead of the ordinary bed rest, it is often found that a wedge-shaped solid pillow is sufficient when placed under the soft pillows; it is of the same width as the mattress, five or six inches in thickness at its thickest end, and about one and a-half or two feet in length. This is supplemented by pillows, so that the patient lies with his head and chest well propped up. The steam-kettle and tent is not always necessary, but when used the supports of the tent are fixed to the floor, and the kettle supported over a gas lamp, supplied by a tube from a neighbouring gas jet. Steam alone is found the most efficacious, and the kettle is carefully watched, so that the supply of water does not run out. On an average the kettle is refilled every three hours, and the temperature kept up to between 60 deg. and 70 deg. F. Some patients have such a strong objection to being shut up in the tent that it is found desirable to keep one side either wholly or partially open, or else to substitute for the tent and kettle a steam inhaler simply. This of course involves more trouble, but is frequently quite satisfactory in the results obtained.

To relieve the violent cough and to render the mucus looser, mist. ipecac. co. is useful (vin. ipecac. ♏x, tinct. camph. co. ♏xx, liq. amm. acetat. ʒiij. aq. ad. ℥i.), but is given with caution, since it contains opium. It is given at first every four hours for four or five doses, then every six hours. Antimony seems to have gone almost out of fashion, though it has lately been used in one or two cases with good results. When the cough is very violent, and the patient is unable to spit up anything, or only very little, bromide of sodium in doses of gr. x. in water (ʒss.) very often quiets the paroxysms and produces sleep. If one dose has no effect, a second is given two hours later, and a third if needful. Poultices are rarely used now as being heavy and messy, but hot fomentations prove just as serviceable. To avoid disturbing the patient as much as possible a many-tailed flannel bandage is placed underneath the chest, and covered by a sheet of oiled muslin, with a good layer of absorbent cotton wool between the two. The fomentation, made of a sheet of absorbent wool, is wrung out at the bedside and immediately slipped beneath the patient. The muslin, wool, and bandage are then closed over the fomentation, and if this has been done successfully it need not be changed for three hours or more. The bandage is fastened loosely to avoid embarrassing the movements of the thorax. To increase the stimulating action of the application turpentine is sometimes sprinkled on the hot wool just before it is put on the chest. If necessary, a smallish linseed and mustard poultice is occasionally put on over the front of the chest when breathing is feeble or pain is great.

Stimulants are usually given in the form of ℥ss. of brandy every two hours till the pulse improves and becomes stronger.

Venesection is occasionally practised, the median basilic being the vein usually selected, and the operation performed in the usual way. One practical point occurs here, viz., the way of holding the basin or other receptacle for the blood to flow into. If the bandage is properly tight above the elbow, then directly the vein is opened the blood spouts out with such force as to miss the basin if simply held under the elbow, and to cover the bedclothes and the dress of anyone standing near. If, however, one basin is held in front of the incision and a second bowl immediately below, the blood stream is arrested by the first and directed by it into the second. Then as the stream gradually loses its force it can be readily caught in the lower basin. The amount withdrawn varies from 8 to 20 ounces, according to the strength of the patient and the effect produced on the radial pulse of the other arm.

Instead of using the drugs mentioned previously, some cases have been treated with very capital results with mist. lobeliæ c. pot. iod. (tr. lobel. aether. ♏xv., pot iod. gr. v., sp. chlorof. ♏x. aq. ad. ℥i.). This mixture given every four hours has a wonderful effect in loosening the cough and rendering the mucus less sticky. As the disease subsides this same mixture is continued three times a day, or, instead, are used either mist. seneg. ammoniat. (ammon. carb. gr. v. tr. scill. ♏xx., infus. seneg. ad. ℥i.), or mist. scillæ (tr. scill. ♏xv., sp. æth. nitr. ♏xxx., liq. amm. acetat. ʒij., syr. limon. ♏xx. aq. ad. ℥i.), each given three times a day. To hasten convalescence, rubbing the chest with various liniments is rather a routine practice. Of the liniments the favourite is one containing ʒiii. each lin. terebinth and liq. ammon. together ol. cajuput ♏v. and ol. olivæ ad. ℥i. The efficacy of this depends greatly upon the energy of the nurse's arms, but when well rubbed in for five minutes or so, night and morning, it gives considerable relief.

Diet is carefully looked after. Milk and soda water being the staple till the acutest stage is past, with occasionally an egg beaten up in milk, or a little custard or arrowroot and mutton broth. After the first few days, if the patient is going on well, fish is given with potatoes, and as convalescence advances, the ordinary meat diet begins.

After the hot fomentations are discontinued as the inflammation goes down, spongiopiline jackets are sometimes used, especially if there is pain in the chest or irritating cough. These jackets require changing twice a day at any rate, and when taken off are scrubbed with a piece of tow or wool dipped in hot water till clean and free from greasiness, and are then well warmed before the fire and put on again. They are readily fastened with tapes or safety pins over each shoulder, and with tapes down one side.

(*b*) *Chronic bronchitis* in one form or another, and associated with various other affections, includes a large proportion of the cases treated in the hospital. In many cases treatment has to be directed to the heart or stomach and digestive organs generally, but in a fair number it is to the lungs that attention is particularly turned. In general the patient comes in with an exacerbation of the chronic complaint, and the various measures and drugs described above are tried. When the acute stage is over there still remain cough, occasional pain in the chest, dyspnœa, and other symptoms which require treatment.

For continual hacking cough with scanty, tenacious sputum, sodium iodide has lately been used with considerable success. It is given in one grain doses dissolved in water (℥ss.), and to start with it is given every hour for 24 hours, then every two hours for another 24 hours, and then every three hours during a similar period. During the night the patient is not rigorously aroused as each dose becomes due, so that an occasional dose is slipped; but with the usual cough in such cases the patient is so liable to be waked up that not many doses are missed. After the third day the drug is given every six hours for a few days and then stopped. By this time there is generally considerable relief; a different medicine is given, such as mist. seneg. ammoniat. or mist. lobel. and pot. iod.

For chronic cases with asthmatic attacks, the pulv. stramon co. is constantly used. It contains pulv.

stramon. ℥i, pulv. semin. anisi et pot. nitr. aa. ℥ss., tabac. contrit. gr. xxx. Of this, as much as will lie on a shilling is burnt, and the smoke inhaled by means of a paper cone or funnel placed over and round the burning powder. This powder, too, is used at times in acute bronchitis to relieve the violent paroxysms of dyspnœa. For these asthmatic cases, as well as for other chronic ones, the mist. stramon. æth. et. opii is useful (tr. stramon ♏xxx., sp. æth. ♏xx., tr. opii ♏iij., aq. camph. ad. ℥j.

In those chronic cases who are also plethoric in habit of body, it is usually necessary to prescribe some purgative mineral water, such as Hunyadi Janos, or for the sake of economy Carlsbad salts are given, one teaspoonful being taken in half a tumblerful of hot water before breakfast. If these fail to produce the desired result mist. alb. is given every morning (mag. carb. levis gr. xxx., mag. sulph. ʒi., aq. menth. pip. ad. ℥i.

Troublesome fits of coughing are often relieved by inhaling steam having tr. benz. co. mixed with it in the proportion of ʒi. of the tincture to ℥i. of boiling water. Other cases get more relief from inhaling vap. conii c. ammon. in steam (succ. conii ʒii., liq. ammon. ♏x.).

In most cases one form or another of linctus is given, chiefly linct us scillæ c opio (oxyme. scill. ♏xxx. tr. camph. co. ♏x., vin. ipecac. ♏v., muscilag. acaciæ. ad. ʒi. When coughing leads to retching or vomiting, or causes much pain, linctus morph. is much used (morph. hydrochlorat. gr. $\frac{1}{16}$, ac. hydrocyan. dil. ♏ij., syr. scill. ♏xx., ac. hydrochlor. dil. ♏j., aq. ad. ʒj). One dose is given as needed, care being taken that not more than four to six doses are given in 24 hours.

To relieve cough, and at the same time aid nutrition, cod liver oil either alone or with extract of malt is continually given. There are very few patients, indeed, who cannot take the oil alone when given after a meal, on milk or lemon water, and until it has had a fair trial there is no hurry to try sedatives. Patients who begin by declaring they cannot take oil, usually manage very well with one teaspoonful once or twice a day at first, and can soon take two teaspoonfuls twice a day.

It is more often in old chronic cases that venesection is used, the indications being cyanosis, bounding full pulse, swollen veins in the neck, and generally a full-blooded habit of body. The Spongiopiline jacket is useful when there is a persistence of wheeziness, with attacks of dyspnœa, and much pain in the chest, and may be worn persistently for two or three weeks. If this is irritating to the skin, or the patient complains of any discomfort, a cotton wool jacket may be substituted.

When indigestion is a prominent symptom the mist. pectoralis acid is useful (acet. scill. ac. sulph. dil., sp. chlorof. aa. ♏x., syr. simpl. ʒi. aq. ad. ℥j), and is much used amongst the out-patients at the City Road Hospital; or an alkaline tonic is tried, such as mist. gent. albi (pot. bicarb. gr. x., tr. zingib ♏j., infus. rhei ʒi. infus. gent. co. ad. ℥j), with tr. nuc. vom. ♏v.; or the mist. calumb. albi (sod. bicarb. gr. x., sp. cholorof. ♏x., inf. calumb. ad. ℥j). As the appetite and digestion improve, so too does the cough go, and the breathing become easier.

In cases of bronchitis with much bad-smelling muco-purulent sputum, or in others with spasmodic pain, we get good results from the mist. ammoniaci et lobel. co. (mist ammoniac ℥gs., vin. ipecac. ♏x., tr. lobel. æth. ♏xij, aq. ad. ℥i), given three times a day. It checks and sweetens the secretion, and eases the cough generally.

The treatment of the heart or kidney symptoms and the like does not come within the scope of this account of treatment, though in many cases of bronchitis their treatment is the main thing to be considered; and especially is this so in alcoholism, where the entire prohibition of alcohol in any form is the first step to be insisted on.

